# Male Rat Offspring Are More Impacted by Maternal Obesity Induced by Cafeteria Diet than Females—Additive Effect of Postweaning Diet

**DOI:** 10.3390/ijms23031442

**Published:** 2022-01-27

**Authors:** Aynaz Tajaddini, Michael D. Kendig, Kelly V. Prates, R. Frederick Westbrook, Margaret J. Morris

**Affiliations:** 1 Department of Pharmacology, School of Medical Sciences, University of New South Wales, Sydney, NSW 2052, Australia; a.seyedtajaddini@student.unsw.edu.au (A.T.); m.kendig@unsw.edu.au (M.D.K.); kvp.86@hotmail.com (K.V.P.); 2 School of Psychology, University of New South Wales, Sydney, NSW 2052, Australia; f.westbrook@unsw.edu.au

**Keywords:** maternal obesity, developmental programming, cafeteria diet, behaviour, liver, gene expression, adiposity, high fat diet

## Abstract

Maternal obesity increases the risk of health complications in offspring, but whether these effects are exacerbated by offspring exposure to unhealthy diets warrants further investigation. Female Sprague-Dawley rats were fed either standard chow (*n* = 15) or ‘cafeteria’ (Caf, *n* = 21) diets across pre-pregnancy, gestation, and lactation. Male and female offspring were weaned onto chow or Caf diet (2–3/sex/litter), forming four groups; behavioural and metabolic parameters were assessed. At weaning, offspring from Caf dams were smaller and lighter, but had more retroperitoneal (RP) fat, with a larger effect in males. Maternal Caf diet significantly increased relative expression of ACACA and Fasn in male and female weanling liver, but not CPT-1, SREBP and PGC1; PPARα was increased in males from Caf dams. Maternal obesity enhanced the impact of postweaning Caf exposure on adult body weight, RP fat, liver mass, and plasma leptin in males but not females. Offspring from Caf dams appeared to exhibit reduced anxiety-like behaviour on the elevated plus maze. Hepatic CPT-1 expression was reduced only in adult males from Caf fed dams. Post weaning Caf diet consumption did not alter liver gene expression in the adult offspring. Maternal obesity exacerbated the obesogenic phenotype produced by postweaning Caf diet in male, but not female offspring. Thus, the impact of maternal obesity on adiposity and liver gene expression appeared more marked in males. Our data underline the sex-specific detrimental effects of maternal obesity on offspring.

## 1. Introduction

Obesity is a global health problem; its prevalence has increased dramatically since 1980, and if this trend continues, most adults worldwide will be overweight or obese by 2030 [1]. Obesity is especially prevalent in developed countries such as the USA [2] and Australia, where over 60% of adults were living with obesity and overweight in 2015 [3]. Women of childbearing age are also increasingly likely to be overweight or obese [4]. Maternal obesity increases the risk of complications such as preeclampsia and gestational diabetes, miscarriage, preterm birth, and neonatal death [5,6,7]. Obesity also influences the development of the placenta, embryo and foetus, leading to long-standing detrimental effects on children’s health [8]. Pregnant women with obesity are more likely to have large for gestational age (LGA) infants, a condition associated with complications such as heart disease, diabetes, and neurobehavioural disorders in adulthood [9,10]. 

There is also a strong association between the risk of childhood obesity and maternal weight gain pre-pregnancy and during gestation [11]. The prevalence of overweight in infancy and childhood have been rising steadily for the last two decades, which, in turn, increases the risk of adult obesity [12]. Fraser et al. [13] reported that children born to mothers with higher gestational weight gain (GWG) had greater adiposity and higher levels of the inflammatory markers c-reactive protein and interleukin 6. Similarly, Russo et al. [14] found a positive association between GWG and offspring obesity and metabolic profile. Specifically, mothers with GWG in the middle and upper tertiles had a 14 and 22% greater risk of having a child with overweight/obesity, respectively. Moreover, glycated haemoglobin (HbA1c) in children was positively associated with maternal GWG. 

There is a strong link between the intrauterine environment and the risk of metabolic diseases such as hyperglycaemia, diabetes, cardiac disorders, and kidney disease in offspring [15]. While conducting prospective human studies of the impact of maternal obesity in humans is of critical importance, there are several constraints in conducting such work. In this regard, work in animal models has confirmed significant effects of maternal obesity on offspring body weight and metabolism [16]. Moreover, studies in animals can aid in identifying mechanisms underlying the effects of maternal obesity on offspring health [17]. A meta-regression study found that animal models of maternal obesity induced by a high fat diet (HFD) consumption had significantly increased body weight, cholesterol, triglyceride, and insulin levels in male and female offspring, while increased blood glucose was only observed in females [18]. Previous work from our lab has shown that overnutrition during gestation and lactation leads to higher adiposity and glucose intolerance in male rat offspring [19] and significant increases in body fat and plasma leptin, insulin, and triglycerides in female rat offspring [20] which were exacerbated by postnatal overnutrition of offspring. 

Several studies have reported on the adverse effects of maternal obesity on the liver health of offspring. It has been shown that offspring born to rat dams fed a high-fat/high-sucrose diet exhibited higher blood glucose and insulin concentrations, increased hepatic triglycerides, and altered gene expression, which may predispose to hepatic steatosis in later life [21]. Bayol et al. [22] showed that offspring exposed to a maternal junk food diet in utero had exacerbated signs of hepatic steatosis and oxidative stress measured in adulthood, compared to offspring from chow fed dams. Dahlhoff et al. [23] found that pre-pregnancy and gestational obesity adversely affected the liver health of adult mouse offspring, and altered gene expression in a sex-specific manner, whereby only male offspring showed overweight, hyperinsulinemia, hyperleptinemia, and hepatic steatosis. However, more information regarding the time-course of the adverse effects of maternal obesity on offspring liver health is required. 

Emerging evidence suggests that maternal obesity may also confer risk of behavioural changes in offspring [24]. A meta-analysis found that children of mothers with obesity, relative to those of normal weight, had 60% higher rates of neurodevelopmental disorders including autism spectrum disorder (ASD) and attention-deficit/hyperactivity disorder (ADHD) [25]. The Infant Feeding Practices Study II found that mothers with a pre-pregnancy BMI > 35 had increased likelihood of having children with psychosocial difficulties (odds ratio (OR): 2.17; 95% CI, 1.24–3.77), ADHD diagnosis (OR: 4.55; 95% CI, 1.80–11.46), and ASD diagnosis (OR: 3.13; 95% CI, 1.10–8.94) [26] at 6 years of age (1311 mother-child pairs). 

Work in animal models has also shown the behavioural effects of maternal obesity on offspring [27]. Sasaki et al. [28,29] found age-specific effects of perinatal HFD exposure: male and female adolescent offspring demonstrated reduced anxiety-like behaviour (testing between postnatal day (PND) 35 and 45), while adult rats exhibited increased anxiety-like behaviour (testing between PND 90 and 110). Graf et al. [30] reported that maternal HFD before and during pregnancy impaired novel object recognition in 16 week old male but not female C57BL/6 J mice offspring consuming a chow diet. In a recent study from our lab, male offspring of lean and obese C57BL/6 J mice dams weaned onto chow underwent behavioural tests from 38 weeks of age; those born to HFD dams had improved sensorimotor gating (PPI) with no significant differences in assessments of cognition, sociability, locomotion, or exploration [31]. 

To date, most work in rodent models of maternal obesity has used purified HFD, and only a limited number of studies have evaluated the effects of ‘cafeteria-style’ diets high in both sugar and fat. Therefore, this study explored the effects of a HFD containing processed, high-energy foods eaten by people presented in a cafeteria format mimicking food choice, along with high sugar (liquid sucrose), on behavioural and metabolic outcomes. Both sexes were studied to examine whether cafeteria diet-induced maternal obesity produced comparable behavioural and metabolic effects in male and female offspring. We also examined the impact of a post-weaning diet switch by weaning offspring onto the same diet as their mother (e.g., chowchow, CafCaf) or onto the other diet (chowCaf, Cafchow) to elucidate the effects of maternal and postnatal diet on offspring health, and whether the two interact to worsen outcomes.

## 2. Results

### 2.1. Dams

#### 2.1.1. Food Intake of Dams

**Figure 1:**
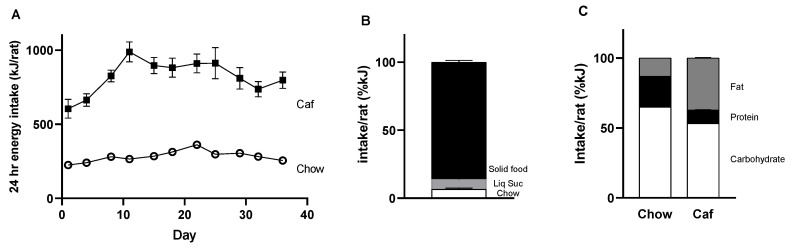

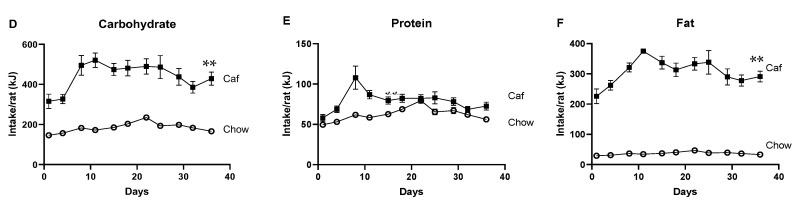
The 24 h energy intake (kJ/rat/day) in chow and Caf rats prior to mating (**A**). Data represent mean ± SEM of 15 chow and 21 Caf females, housed three per cage. Intake of diet component subgroups (chow, solid Caf, and liquid sucrose) in Caf females as percent total kJ is shown in (**B**). Overall macronutrient intake in chow and Caf groups (**C**) as well as macronutrient intake over time (**D**–**F**) is shown. Data were analysed by mixed ANOVA. ** *p* < 0.01, significant effect of Caf diet.

Total 24 h energy intake during the five weeks before mating is shown in Figure 1. Overall, female rats fed the Caf diet consumed almost three times as much energy as those fed chow (Figure 1A); mean food intake over the five week period was 283 ± 1 kJ/day for the chow group and 820 ± 4 kJ/day for the Caf group (*p* < 0.05). 

As shown in Figure 1B, the Caf group derived most energy from solid cafeteria diet foods (86%), followed by liquid sucrose (7%), and then chow (7%). When expressed as a percentage of total energy, the Caf group derived a higher proportion of energy from fat and less from protein and carbohydrate than the control rats (Figure 1C). These proportions were consistent across the five weeks of diet (data not shown). 

**Figure 2:**
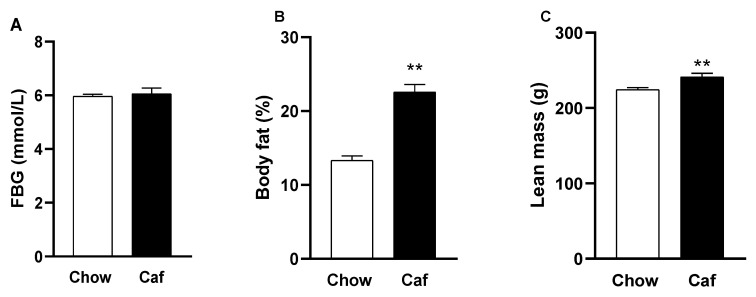
Fasting blood glucose (FBG) (**A**), body fat percentage (**B**) and body lean mass (**C**) of females at days 30 and 31 of the diet, respectively. Data represent mean ± SEM of 11 chow and nine Caf females and were analysed by independent Student’s *t*-test or Mann–Whitney U test. ** *p* < 0.01, significant effect of the Caf diet.

In terms of macronutrient intake, the Caf group consumed significantly more carbohydrate than the chow group (F = 50.80, *p* < 0.001), which varied over time (F = 15.11, *p* < 0.01), but the time by group interaction was not significant (F < 1.0) (Figure 1D). Protein intake was significantly higher in the Caf than chow group (F = 10.69, *p* < 0.05), with no significant effect of time (F = 2.78, *p* = 0.13), while time by group interaction was significant (F = 6.09, *p* < 0.05) (Figure 2E). The Caf group consumed significantly more fat than the chow group (F = 161.32, *p* < 0.001), which did not significantly vary over time (F = 2.14, *p* < 0.18), and there was no time by group interaction (F < 1.0, *p* < 0.46) (Figure 2F). 

#### 2.1.2. Fasting Blood Glucose and Echo-MRI

Fasting blood glucose, measured on day 30 of the diet, was not significantly different between groups (Figure 2A). Percent body fat and lean mass were significantly higher in the Caf than chow group (Mann–Whitney U test, both *p* < 0.01) (Figure 2B,C). 

#### 2.1.3. Body Weight 

**Figure 3:**
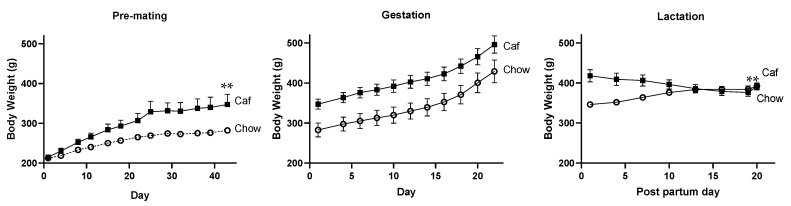
Body weight over time in female rats consuming chow or Caf diet, before mating, during gestation and lactation. Data are expressed as mean ± SEM of 11 chow and nine Caf females. Data were analysed by mixed ANOVA. ** *p* < 0.01, significant time × maternal diet interaction.

Figure 3 shows that prior to mating, all animals gained weight over time (F = 709.64, *p* < 0.001) with a significant time × diet interaction (F = 89.01, *p* < 0.001), indicating greater weight gain in the Caf than chow group. Mean percent body weight gain over this time was 67% for the Caf group and 30% for the chow group. 

Both Caf and chow groups gained weight during gestation, indicated by a significant effect of time (Figure 3; F = 918.51, *p* < 0.001). The rate of this increase did not differ between groups, with no group × time interaction (F < 1.0, *p* = 0.63). At the end of gestation, the Caf group weighed 20% more than the chow group (*p* < 0.01, main effect of diet). Interestingly, during lactation, body weight followed different patterns in the Caf and chow groups (Figure 3), while the Caf group lost weight, the chow group gained weight, so that groups converged at post-partum day 13. This was reflected in a significant time by group interaction (F = 64.95, *p* < 0.001) with no main effect of time (F < 1.0, *p* = 0.35). At the end of lactation, body weight did not differ between groups (t = 0.225, *p* = 0.825). 

#### 2.1.4. Birth Characteristics 

As shown in Table 1, there were no significant differences in litter size or male/female ratio between litters born to Caf and chow dams; however, the average body weight of male offspring from Caf litters was significantly lower than those from chow litters (t = 2.89, *p* < 0.01). 

#### 2.1.5. Dam Endpoint Measures

Table 2 shows the endpoint measures collected in dams at three weeks post-partum. At that time, no differences were observed in body weight, length, and girth, but RP fat mass was approximately doubled in the Caf group. There was no significant difference in blood glucose and liver weight, while cecum weight was significantly lower in Caf relative to chow dams (Table 2). 

### 2.2. Offspring 

#### 2.2.1. Pre-Weaning 

**Figure 4:**
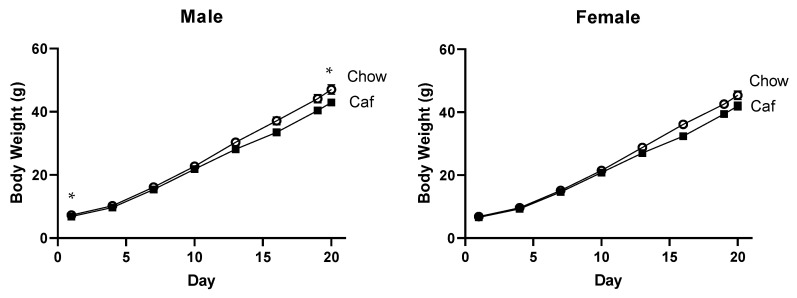
Offspring body weight across the lactation period in 21 females (14 chow, 7 Caf) and 23 males (13 chow, 10 Caf). Data are expressed as mean ± SEM of the average weight of litter and were analysed by mixed ANOVA. * *p* < 0.05, significant effect of maternal Caf diet.

Figure 4 shows the body weight of offspring from Caf and chow fed dams during lactation; males from Caf dams were significantly smaller at birth (see Table 1; t = 2.70, *p* < 0.05). The marked offspring weight gain across lactation (F = 1609, *p* < 0.001) was not affected by maternal diet (F = 2.26, *p =* 0.15). At weaning, male offspring from Caf dams remained significantly lighter (t = 2.36, *p* < 0.05). In females, body weight significantly increased over time (F = 1854.97, *p* < 0.001) with no diet × time interaction (F = 2.16, *p* = 0.158). 

Table 3 shows endpoint measures in a subset of chow and Caf offspring collected at weaning (PND 20). Offspring born to Caf dams exhibited significantly lower body weight, nasoanal length, liver mass, kidney mass, and blood glucose, relative to offspring from chow dams (maternal diet main effects, all *p* < 0.05). There was a trend toward smaller girth in Caf dam offspring (*p* = 0.051, maternal diet main effect). Retroperitoneal fat differed according to maternal diet (*p* < 0.01), sex (*p* < 0.01) with a maternal diet × sex interaction (*p* < 0.05), suggesting that maternal Caf diet increased RP fat mass more in males than females (see Table 3). No other main or interaction effects were significant. 

#### 2.2.2. Post-Weaning Measures

##### Food Intake

**Figure 5:**
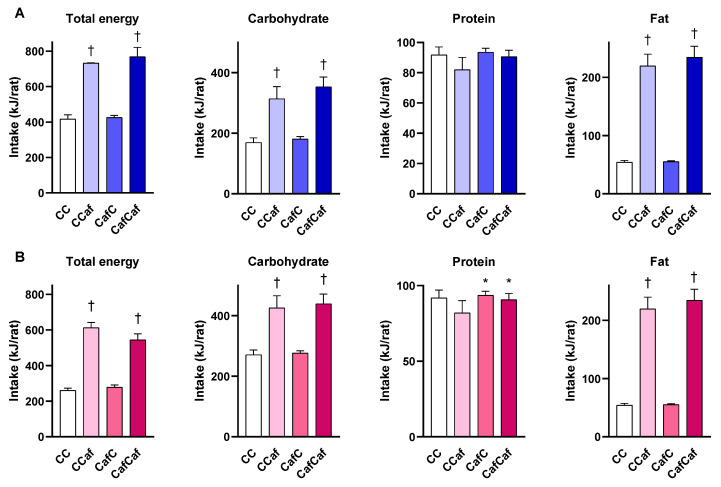
The 24 h food consumption and macronutrient intake of male (**A**) and female (**B**) offspring at seven weeks of age. ChowChow (11 male, 11 female), ChowCaf (11 male, 11 female), CafChow (13 male, 11 female), and CafCaf (13 male, 13 female). Data are expressed as mean ± SEM and were analysed by two-way ANOVA. * *p* < 0.05 significant effect of maternal Caf diet, † *p* < 0.05 significant effect of the offspring postweaning diet.

Figure 5 shows the macronutrient intake of male (A) and female (B) offspring when measured at seven weeks of age. Maternal diet significantly increased protein intake of female offspring from Caf mothers (F = 4.32, *p* < 0.05). There was no significant effect of offspring diet on protein intake in females. Postweaning exposure to Caf diet significantly increased total energy, carbohydrate, and fat intake in both males and females. 

##### Body Weight 

**Figure 6:**
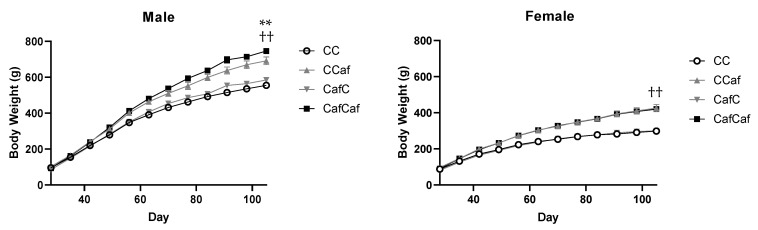
Body weight of female and male offspring post weaning. ChowChow (11 male, 11 female), ChowCaf (11 male, 11 female), CafChow (13 male, 11 female), CafCaf (13 male, 13 female). Data are expressed as mean ± SEM and were analysed by mixed ANOVA. ** *p* < 0.01 significant time × maternal diet interaction, †† *p* < 0.01 significant time × offspring diet interaction.

After weaning, body weight in male offspring increased, as expected (linear effect of time: F = 71.63, *p* < 0.001), moreover, this was significantly accelerated by maternal obesity (time × maternal diet interaction: F = 8.66, *p* < 0.01; Figure 6), and postweaning exposure to the Caf diet (F = 52.08, *p* < 0.001), with no 3-way interaction between time, maternal, and offspring diet (F < 1.0, *p* = 0.591). 

In female offspring, body weight of rats consuming Caf was higher than the chow group (F = 90.42, *p* < 0.001), regardless of their maternal diet, with no mother × offspring diet interaction (F < 1.0, *p* = 0.792). After weaning, average body weight was increased by Caf consumption, as expected (F = 121.17, *p* < 0.001). There was no maternal diet effect on body weight in females (F < 1.0, *p* = 0.406), and no interaction (F < 1.0, *p* = 0.726; Figure 6). 

##### Fasting Blood Glucose and Echo-MRI

**Figure 7:**
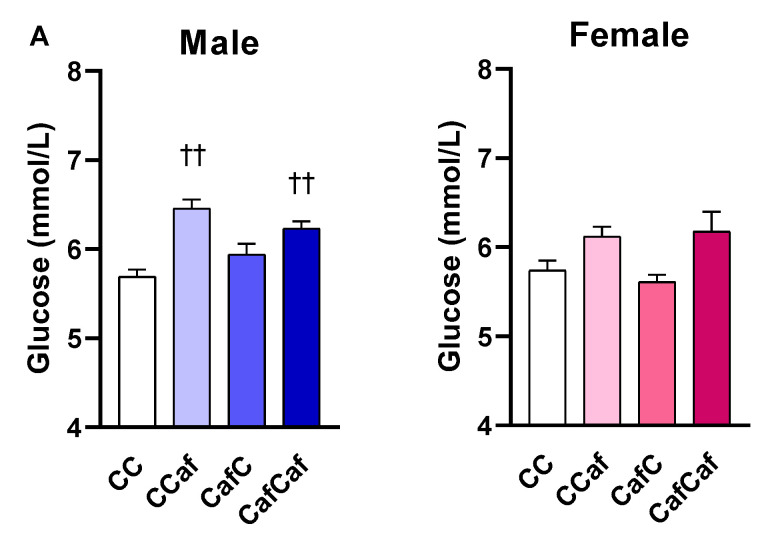

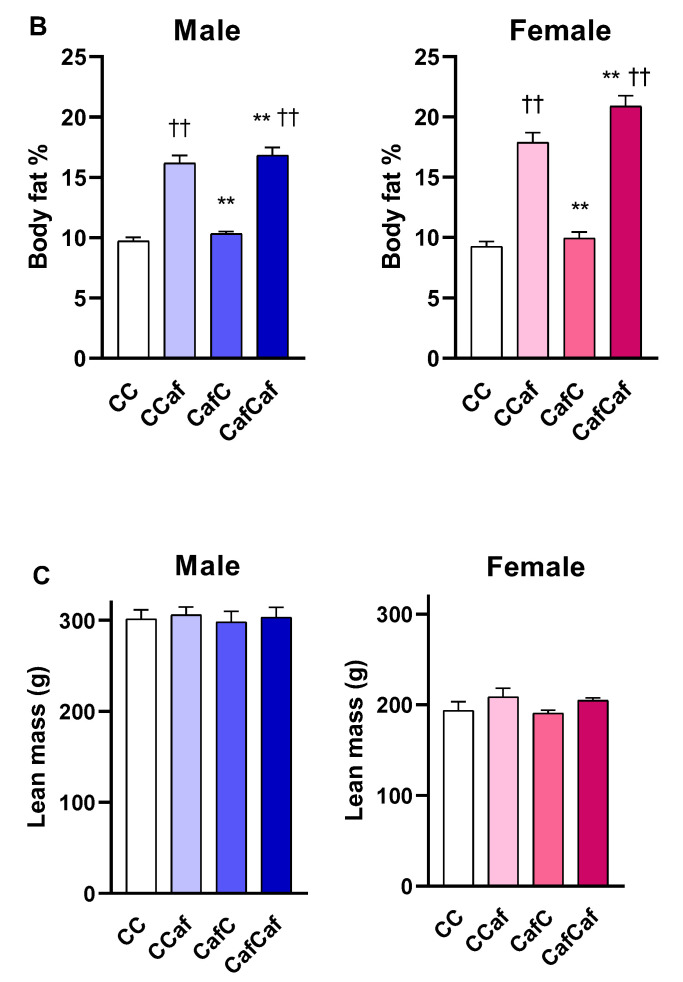
Fasting blood glucose concentration (**A**), body fat % (**B**) and lean mass (**C**) of male and female offspring at seven weeks of age. ChowChow (11 male, 11 female), ChowCaf (11 male, 11 female), CafChow (13 male, 11 female), and CafCaf (13 male, 13 female); C depicts chow. Data are expressed as mean ± SEM and were analysed by three-way ANOVA. ** *p* < 0.01 significant effect of maternal Caf diet, †† *p* < 0.01 significant effect of offspring post weaning diet.

Caf-fed male offspring had significantly higher fasting blood glucose levels relative to the chow fed males (F = 14.01, *p* < 0.01; Figure 7A). The EchoMRI tests in week 7 showed that both maternal and offspring Caf diet significantly increased body fat percent in males and females (Figure 7B). There was a significant maternal diet × sex effect in lean mass (*p* < 0.05). However, there was no significant effect of maternal or offspring diet in lean mass of either sex (Figure 7C). 

##### Elevated Plus Maze (EPM) Behaviour

**Figure 8:**
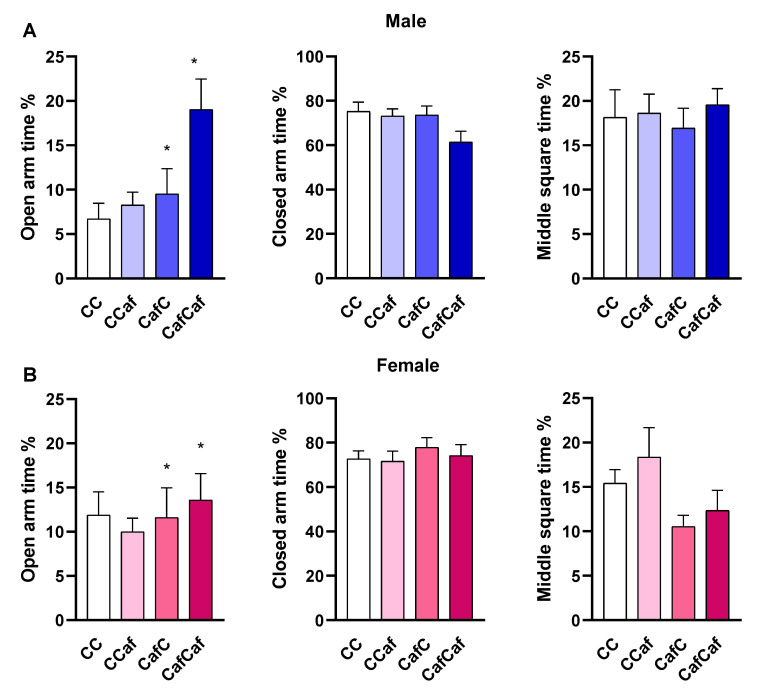
Elevated plus maze test results in male (**A**) and female (**B**) offspring. ChowChow (11 male, 11 female), ChowCaf (11 male, 11 female), CafChow (13 male, 11 female), and CafCaf (13 male, 13 female); C depicts chow. The percentage of time spent in open and closed arms, and in the middle square were determined for each animal over a 5 min session. Data are expressed as mean ± SEM and were analysed by three-way ANOVA. * *p* < 0.05 significant effect of maternal Caf diet.

Percent time in the open arms of the maze was significantly increased by maternal exposure to Caf diet (F = 5.84, *p* = 0.018) with no other significant effects of postweaning diet (F = 2.18, *p* = 0.14), sex (F < 1), or any interactions (all *p* > 0.05; Figure 8).

##### Place/Object Test Behaviour

**Figure 9:**
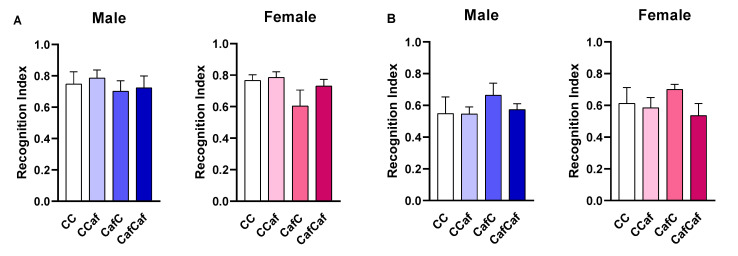

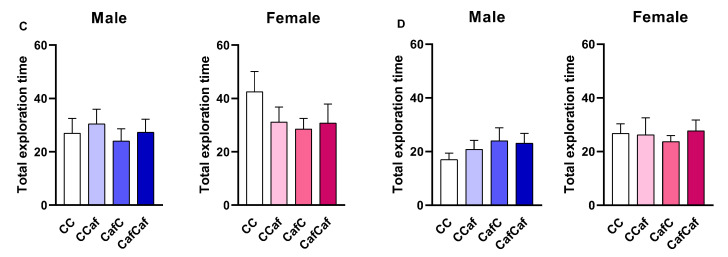
Object (**A**) and place (**B**) task test in male and female offspring. ChowChow (11 male, 11 female), ChowCaf (11 male, 11 female), CafChow (13 male, 11 female), and CafCaf (13 male, 13 female); C depicts chow. The recognition index was calculated for each animal based over a 10 min session. Total exploration time for object (**C**) and place (**D**) task tests was calculated. Data are expressed as mean ± SEM and were analysed by two-way ANOVA.

Our results showed no significant effect of maternal or offspring diet on object or place recognition tasks, nor in total exploration time (Figure 9). 

##### Endpoint Measures in 14 Week Old Adult Offspring

Table 4 summarises the endpoint measures collected in 14 week old offspring. Significant maternal diet × sex interactions were found for RP fat mass (F = 8.39, *p* = 0.005), liver mass (F = 10.92, *p* = 0.001), and terminal body weight (F = 6.97, *p* = 0.01), indicating distinct effects of maternal obesity in males and females for these measures, regardless of postweaning diet. Subsequent analyses within each sex showed that in males, maternal Caf diet exposure significantly increased body weight, girth, liver weight, and RP fat mass in male offspring (see Table 4), while postweaning Caf diet significantly increased all end point measurements except for blood glucose and tibia length (Table 4). In contrast, in females, there were no significant effects of maternal diet, and no maternal × postweaning diet interactions (all *p* > 0.05), though postweaning Caf diet exposure significantly increased all measurements except for tibia length (Table 4). Analyses also found significant sex differences in terminal body weight (F = 656.29, *p* < 0.001), nasoanal length (F = 761.45, *p* < 0.001), girth (F = 249.14, *p* < 0.001), liver mass (F = 492.62, *p* < 0.001), heart weight (F = 198.83, *p* < 0.001), RP fat (F = 130.51, *p* < 0.001), and tibia length (F = 417.45, *p* < 0.001), but not blood glucose (F = 3.37, *p* = 0.07).

##### Plasma Measures in 14 Week Old Adult Offspring 

**Figure 10:**
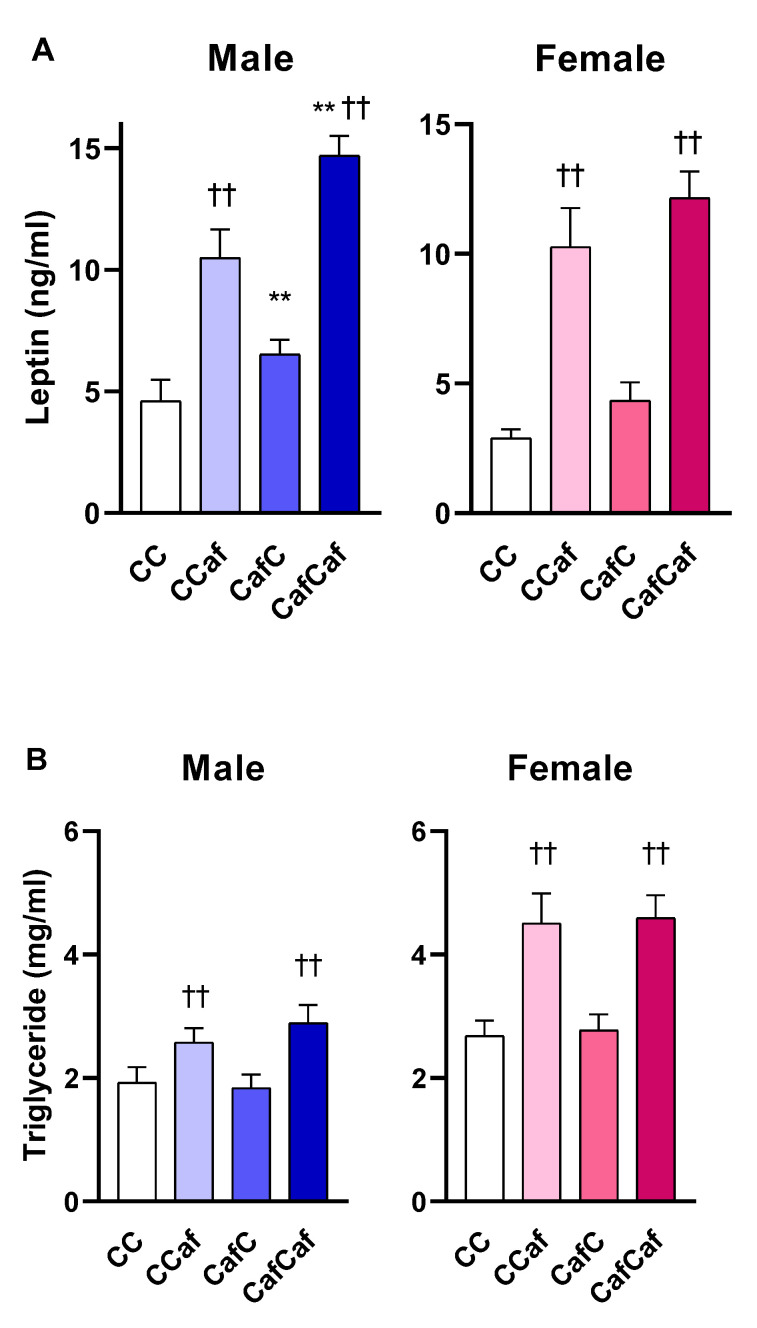

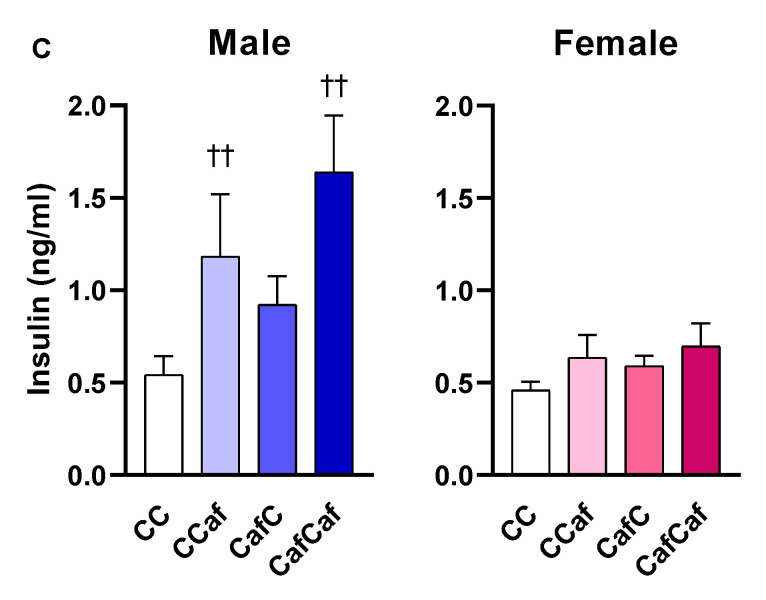
Plasma leptin (**A**), triglyceride (**B**), and insulin (**C**) levels in male and female offspring at 14 weeks. ChowChow (11 male, 11 female), ChowCaf (11 male, 11 female), CafChow (13 male, 11 female), and CafCaf (13 male, 13 female); C depicts chow. Data are expressed as mean ± SEM and were analysed by two-way ANOVA. ** *p* < 0.01 main effect of maternal Caf diet, †† *p* < 0.01 main effect of offspring postweaning diet.

Maternal Caf diet consumption significantly increased offspring plasma leptin (F = 13.28, *p* = 0.001), but not triglyceride (F = 0.197, *p* = 0.659) concentrations, with higher concentrations in male offspring. As expected, postweaning Caf diet increased plasma leptin (male; F = 64.49, *p* < 0.001, female; F = 58.00, *p* < 0.001) and triglycerides (male; F = 11.21, *p* = 0.002, female; F = 26.04, *p* < 0.001), in both sexes. Maternal diet did not modulate leptin or triglyceride concentrations in female offspring. There were postweaning diet effects on insulin in male (F = 7.54, *p* < 0.01), but not female (F = 3.46, *p* < 0.06; Figure 10) offspring.

##### Liver Gene Expression in Weanling (PND 20) and Adult Offspring (14 Weeks)

**Figure 11:**
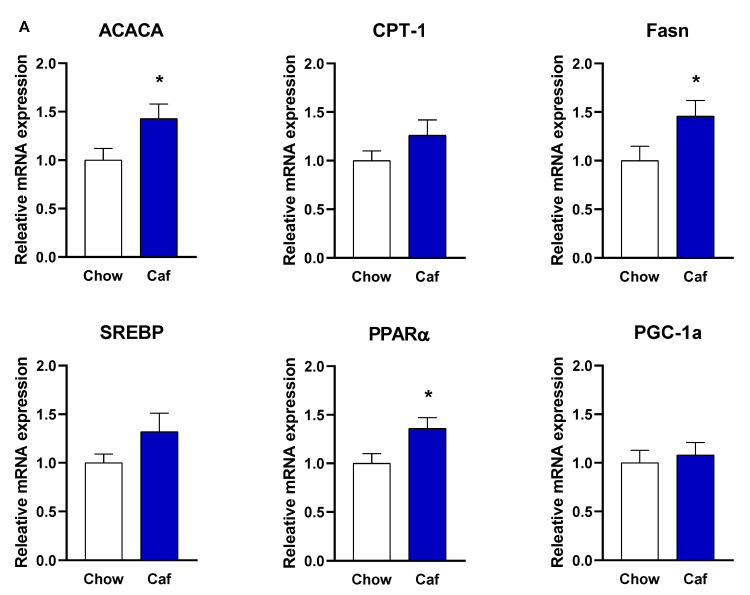

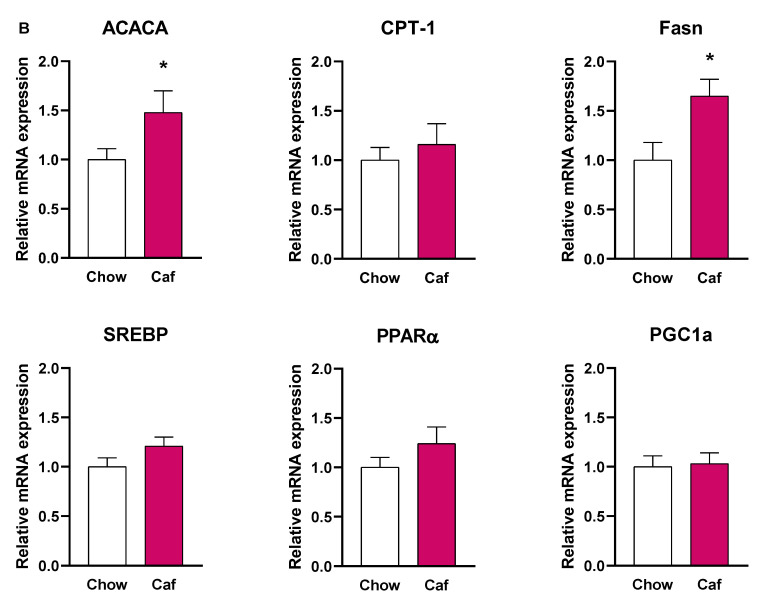
Liver ACACA, CPT1, Fasn, SREBP, PPARα, and PGC1a mRNA expression in males (**A**) and females (**B**) at PND 20, in Chow (11 male, 12 female) and Caf (10 male, 7 female) offspring. Data are expressed as mean ± SEM and were analysed by independent *t*-test. * *p* < 0.05 main effect of maternal Caf diet.

In offspring at weaning, maternal exposure to the Caf diet significantly increased the relative expression of liver ACACA (male; t = 2.25, *p* < 0.05, female; t = 2.16, *p* < 0.05) and Fasn (male; t = 2.09, *p* < 0.05, female; t = 2.51, *p* < 0.05), but not CPT-1, SREBP, and PGC1 (all *p* > 0.05). PPARα relative expression was significantly elevated in male (t = 2.42, *p* < 0.05), but not female weanlings born to Caf dams (t = 1.29, *p* > 0.05) (Figure 11). 

**Figure 12:**
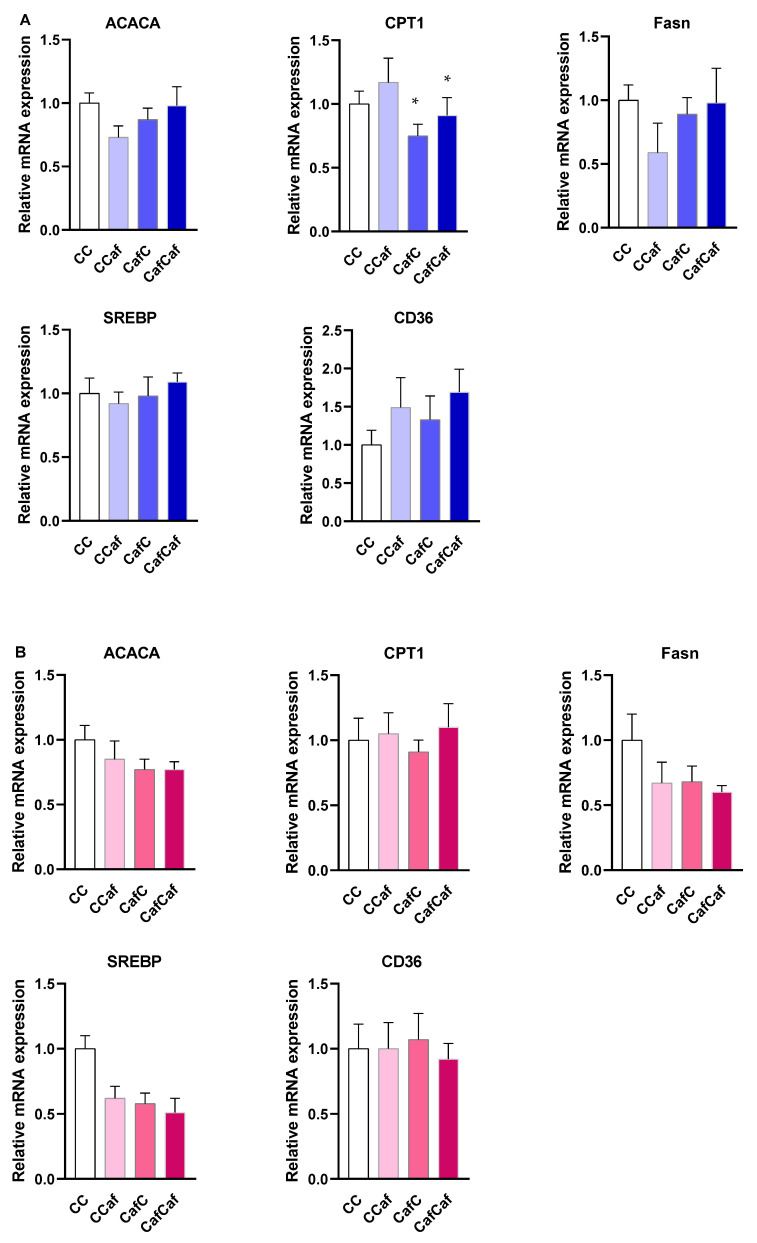
Liver ACACA, CPT1, Fasn, SREBP, and CD36 mRNA expression in males (**A**) and females (**B**) at 14 weeks of age. ChowChow (11 male, 11 female), ChowCaf (11 male, 11 female), CafChow (13 male, 11 female), and CafCaf (13 male, 13 female); C depicts chow. Data are expressed as mean ± SEM and were analysed by two-way ANOVA. * *p* < 0.05 main effect of the maternal Caf diet.

A distinct pattern was observed in the livers of adult (14 week) offspring. Here, male offspring born to Caf fed dams showed significantly lower relative expression of CPT-1 (F = 6.13, *p* < 0.05), but no significant changes in ACACA, Fasn, SREBP, and CD36 (all *p* > 0.05). There was no significant effect of maternal diet on liver gene expression in females. Moreover, no significant effect of postweaning diet on liver gene expression was observed in either sex (Figure 12). 

## 3. Discussion

Human and animal studies have reported that maternal obesity during gestation and lactation has detrimental effects on offspring health such as increased risk of obesity, metabolic syndrome, type 2 diabetes, and cardiovascular disease [15,16]. This study examined the interactive effects of maternal and offspring exposure to a palatable Caf diet on metabolic and growth parameters, liver gene expression, and cognitive function. The key findings were that offspring born to Caf dams had elevated adiposity—particularly males—despite being lighter and smaller than chow offspring. Elevated adiposity in male offspring from Caf dams persisted into adulthood, when these offspring presented as significantly heavier, with greater RP fat, liver mass, and plasma leptin than males born to chow dams. Young adult offspring from Caf dams exhibited less anxiety, but did not differ in the measure of short-term memory. Male and female weanling rats born to Caf dams had increased expression of hepatic ACC and FAS, while in adulthood, we found decreased CPT-1 expression in male offspring. 

Human studies have shown that higher maternal body weight is related to increased BMI and percentage body fat in childhood and adulthood [32]. Boys born to obese mothers showed increased body fat from two to six years of old, while there was no significant difference in body fat of girls born to obese mothers during the first six years of life [33]. Moreover, obese women are at higher risk of delivering small for gestational age (SGA) children. A Dutch study of 385 women with obesity reported an 18.8% incidence of SGA infants compared to 10% in the normal population [34]. Animal studies have also reported varied effects of maternal obesity on the body weight of male and female offspring during development [18]. While the effects were modest, we found significant effects of maternal Caf diet on offspring body weight in early life, so that males born to Caf dams were lighter at birth and both sexes were significantly lighter at weaning, despite having elevated adiposity. This aligns with previous research demonstrating that offspring born to obese dams exhibit a ‘thin outside, fat inside’ phenotype [35]. Intriguingly, by adulthood, male offspring from Caf dams had a higher body weight, RP fat, liver mass, and plasma leptin than those born to chow dams. Our results are in line with Mucellini et al. [36], who reported that male rats born to dams consuming a Caf diet during gestation and lactation had similar body weights as the chow group as juveniles (PND 30), but greater body weight as adults (PND 120). Pomar et al. [35] reported that both male and female rat offspring of dams fed the cafeteria diet only during lactation had lower body weight and lean mass, but greater adiposity at weaning and at three months of age, maternal cafeteria diet significantly increased body fat content of offspring in both sexes in comparison with the controls. By six months of age, offspring of Caf fed dams still had higher body fat content, but the difference was not statistically significant.

Several factors may underlie the effects of maternal obesity observed here; at the time of birth, our Caf mothers were heavier than the chow dams, although this cohort lost some weight during lactation, which we have not previously observed. We did not measure energy intake during lactation in order to avoid disturbing the dams. It is known that an unbalanced diet in mothers (lower protein and higher fat) may alter milk composition, in turn reducing growth capacity and lean mass gain in offspring [35]. Indeed, this may explain the increased protein intake observed in female offspring from Caf-fed dams at seven weeks of age in the present study. Alternatively, decreased lean mass in Caf offspring may relate to altered nursing behaviour and mammary gland metabolism in the dams [37]. Moreover, studies have shown that higher leptin levels in Caf diet-fed dams can prevent increases in energy intake and body weight of offspring [38,39]. This theory is in line with studies that reported a higher possibility of mothers with obesity giving birth to children with lower body weight and increased risk of metabolic syndrome and cardiovascular disease in adulthood [40,41].

Our results indicated sex-specific effects of maternal obesity on offspring metabolic health, with male offspring appearing to be more strongly affected by maternal obesity than females on measures of liver mass, RP fat mass, and terminal body weight. While the biological significance of these findings is unclear and more work is required, studies in humans also show sex-dependent effects of maternal diet on offspring. For example, Francis et al. studied 761 mother–offspring pairs to assess the relationship between the Healthy Eating Index (HEI) in pregnancy and offspring body weight, fat mass, and metabolic measures. Results showed an inverse correlation between maternal HEI and glucose, insulin, HOMA-IR, and adiponectin in male, but not female children [42]. Animal studies from our laboratory have also shown that maternal obesity is associated with glucose intolerance, hyperinsulinemia, increased leptin level, and hyperphagia in male rat offspring [43]. A recent study found that HFD induced maternal obesity increased adiposity more strongly in female rats at weaning, but was more persistent in males in later life, potentially linked to lasting changes in adipocyte size in males [44]. Some of the differences between sexes may relate to sex hormones, and oestrogen exerts protective effects through actions on adipose, skeletal muscle, liver, and pancreatic β-cells. Thus, oestrogens have been shown to protect against HFD-induced insulin resistance and glucose intolerance in mice [45] and work in oestrogen receptor-α (ERα) knockout mice suggests that oestrogen may improve hepatic insulin sensitivity through ER-α signalling [46]. Differences in placental growth and nutrient transfer may also contribute to sex-specific changes in offspring [47]. 

Moreover, Dudele et al. [48] found that maternal exposure to HFD or lipopolysaccharide infusion during pregnancy and lactation led to sex dependent effects in mouse offspring. The proinflammatory response to HFD was more pronounced in male offspring at adulthood, while female offspring showed larger metabolic effects. Krus et al. [49] also showed that male mouse offspring from dams fed HFD during gestation had higher body weight and larger adipocytes after being re-exposed to HFD at 26 weeks of age. They were also glucose intolerant and insulin resistant, with mitochondrial dysfunction and hepatosteatosis. These results align with the present study by showing that maternal HFD exposure exacerbates the detrimental response to a HFD challenge in male offspring later in life. 

While we observed no maternal diet effect on offspring blood glucose, postweaning cafeteria diet exposure increased blood glucose levels at seven and 14 weeks in male and female offspring, respectively. This is similar to the results of Parente et al. [50], who reported increased blood glucose in the offspring of both sexes at six months with postweaning HFD compared to chow fed rats (male offspring were more affected than females). However, Mucellini et al. [36] found no significant effects of maternal or postweaning cafeteria diet on male offspring blood glucose at puberty (30 d) whereas there was a significant increase observed in blood glucose, insulin, and leptin levels in male offspring in adulthood (120 d). However, further investigation of the impact on glycaemic control by glucose tolerance tests would be useful.

In this study, we found that maternal obesity altered plasma leptin, but not triglycerides, with higher concentrations in male offspring from Caf dams. As expected, postweaning Caf diet increased plasma leptin and triglycerides in both sexes. Maternal diet did not modulate leptin or triglycerides in adult female offspring. Intriguingly, postweaning exposure to a Caf diet increased insulin in male but not female offspring. Clinical studies have reported that young adults (especially male offspring) born to obese mothers have increased glucose and insulin concentrations and higher leptin levels compared to children of lean mothers [51]. Our lab [43] also previously found that HFD-induced maternal obesity led to hyperleptinemia, hyperinsulinemia, and hyperphagia in offspring, which was aggravated by postweaning HFD. Maternal HFD also elevated plasma leptin, an effect that was aggravated by post weaning HFD [20]. Parente et al. [50] reported that maternal and postweaning HFD altered carbohydrate metabolism, with significant effects on serum leptin level, fat mass, and adipocyte size. 

Male and female weanling rats had increased hepatic ACC and FAS (key enzymes in the fatty acid synthesis pathway) expression in response to maternal Caf diet exposure. In line with observations from this study, offspring hepatic FAS expression has been reported to increase with maternal obesity in mouse models [52,53]. Despite increased expression of ACC and FAS, SREBP, a known regulator of ACC and FAS expression, was not altered by maternal Caf diet, which might be due to the regulating role of other factors including the cAMP-dependent kinase (PKA) pathway, 5 AMP-activated protein kinase (AMPK), or insulin signalling pathways. The only suggestion of sex-specific effects was in PPARα expression, which was increased in male but not female weaners. The relative expression of CPT-1, a target gene of PPARα, was modestly increased, but not significantly different. Our findings align with those of Giudetti et al. [54], who reported that maternal Caf diet increased CPT-1 expression in male rats at PND 40, and Kjaergaard et al. [21], who showed that high fat/high sucrose diet during gestation and lactation increased CPT1 expression with no significant effect on PPARα and CD36 in rat offspring at weaning. 

To complement the changes observed in hepatic gene expression at weaning, we examined hepatic gene expression in adults. In adult male offspring, maternal Caf diet decreased CPT-1 expression, however, the effects on other genes and in female offspring were not significant. Daniel et al. [55] showed decreased expression of PPARα and CPT1 in 13 week old rat offspring born to Caf diet fed dams. Other mouse studies have found that maternal HF diet decreased PPARα and CPT1 relative expression in offspring at PND 10 [52] and 12 weeks of age [53]. In contrast, another mouse study found that hepatic CPT1 expression in 15 week old offspring born to dams consuming a HF diet during gestation and lactation was unchanged [56]. 

The present study observed that seven week old offspring from Caf dams spent more time in the open arms of the EPM (indicative of less anxiety) than those born to chow dams, regardless of their postweaning diet. While this effect appeared stronger in males, the sex by maternal diet interaction was not statistically significant. Wright et al. [57] found that maternal Caf diet provided only during lactation reduced anxiety-like behaviour in the EPM and open field (OF) tests in adult rat male and female offspring tested at 10 weeks of age. Speight et al. [58] showed that 23 day old offspring born to dams consuming a Caf diet during lactation had less anxiety in an OF test, which may be influenced by diet-induced differences in maternal nursing behaviour and licking, which can influence anxiety levels in offspring. Sasaki et al. [28,29] also found reduced anxiety in adolescent rats with perinatal HFD exposure, whereas adult rats exhibited increased anxiety. Curi et al. [59] showed that maternal HFD increased anhedonic behaviour in male mice offspring, as indicated by decreased sucrose preference, and reduced anxiety-like behaviour in the EPM, compared to offspring born to chow dams. Thus, our data are in line with previous literature and suggest that maternal obesity tends to reduce anxiety in offspring, though effects may be sex- or age-dependent. 

The relationship between obesity, diet, and anxiety is complex. While meta-analytic evidence suggests that obesogenic diets increase anxiety-like behaviour in the EPM and OFT in rodents consuming such a diet [60], here, we found anxiolytic effects of maternal Caf diet exposure in young offspring, regardless of whether these offspring were themselves fed a chow or Caf diet. Altered maternal care by Caf dams, maternal obesity-induced changes to offspring brain development, inflammatory changes, and maternal transfer of gut microbiota are among the mechanisms that may mediate behavioural changes in offspring [20,24].

We found no significant effects of maternal or postweaning Caf diet on short-term recognition memory of the offspring. While some previous studies have reported no effect of maternal HFD exposure on mouse offspring at 38 weeks of age [31], Graf et al. [30] showed sex-dependent effects of maternal HFD in mice offspring weaned to a chow diet at 16 weeks of age, with HFD exposure impairing novel object recognition in male but not female offspring. Similarly, maternal HFD in a rat model resulted in impaired object recognition and spatial memory in adult male offspring (PND 95) compared to the offspring of chow fed dams [61]. Hence, the effects of high-energy diets on cognition are multifactorial and might depend on the composition of the diets, their duration, and the age at which they are introduced as well as the age of testing [24].

## 4. Materials and Methods

### 4.1. Animals and Diet

**Figure 13:**
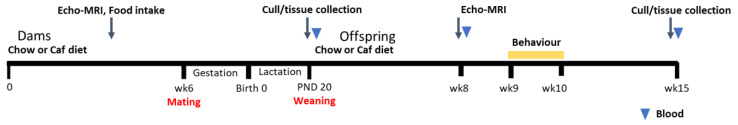
Experimental design. After exposure to chow or a Caf diet for six weeks, female rats were mated with lean male rats, maintained on their diet throughout gestation and lactation and then euthanised three weeks post-partum. Offspring were weaned onto chow or Caf diet, forming four groups (ChowChow, ChowCaf, CafChow, CafCaf), where the first word refers to maternal diet and the second to offspring postweaning diet. Tissues were collected from male and female offspring at PND 20 and 15 weeks to examine the effects of maternal obesity. Offspring underwent Echo-MRI scanning (fat/lean mass) at eight weeks of age, and behavioural tests at 9–10 weeks of age.

This study was approved by the Animal Care and Ethics Committee at UNSW Sydney (approval #19/74A) and conformed to the Australian code for the care and use of animals for scientific purposes 8th edition (2013). Adult female (*N* = 36) and male (*N* = 16) Sprague-Dawley rats were obtained from the Animal Resources Centre (Perth, Australia) and were housed three per cage at 18–22 °C on a 12-h light/dark cycle (lights on 3:00 a.m.–3:00 p.m.). After one week of acclimatisation, mean body weight of female rats was 215 ± 1 g (SEM). Females were then divided into two groups fed either standard rat chow (59% carbohydrate, 26% protein, 15% fat) (*n* = 15, mean weight = 213 ± 2 g [SEM]) or a cafeteria (*n* = 21, 217 ± 1 g) diet comprising chow plus a combination of foods high in fat and/or sugar (biscuits, cakes, pies, dim sims, and 10% sucrose solution). All rats were provided with potable water ad libitum. A timeline of the experiment is provided in Figure 13.

### 4.2. Food Intake, Body Composition, and Plasma Glucose Measures in Dams 

Prior to mating, food intake over a 24 h period was measured twice per week as described previously [62]. Rats were moved to a fresh cage at the beginning of food intake measures, which used the same set of foods. Each food item was weighed to the nearest 0.1 g and was collected and re-weighed 24 h later. The difference in weight was used to calculate energy intake (kJ) using information from the manufacturer. Energy intake was analysed on a per-cage basis. 

After 30 days of diet, female rats underwent EchoMRI-900 (EchoMRI LLC, Houston, TX, USA) at BRIL, UNSW. Fat and lean mass were quantified after weighing each rat to the nearest 0.1 g. Rats were scanned individually (approx. 2 min/rat). Fasting glucose was measured in blood collected from the tail at 32 days of diet, after an overnight fast using a glucometer (Accu-Check^®^ Performa II blood glucose meter, Roche, Germany). An additional 100 µL blood was collected into a sterile tube containing EDTA and centrifuged (5 min, 6000 RCF, 4 °C). Plasma was stored at −30 °C.

### 4.3. Mating and Lactation 

After six weeks of diet, females were mated with chow fed males by co-housing 2–3 females and one male for five days. At the time of mating, mean body weight in chow and Caf groups was 285 ± 4 g and 347 ± 11 g, respectively (t = 10.61, *p* < 0.001). After five days, males were removed, and females were weighed every 2–3 days thereafter. Pregnancy was inferred based on weight gain and females were housed individually from approximately gestation day 16. Dams and offspring were weighed every three days during lactation, commencing on PND 1. Litters were standardised to six male and six female pups, where possible. To avoid excessive interference with the cage bedding and any associated stress, food intake was not measured during lactation. Caf foods were placed in the food hopper, rather than on the cage floor after PND 13, when offspring were observed moving around the cage and searching for food.

### 4.4. Weaning Tissue Collection and Diet Allocation

On PND 20, one male and one female pup per litter were anaesthetised by i.p. injection of ketamine/xylazine, and then decapitated. Liver, RP fat, and plasma were collected, and the cecum was weighed. Remaining male and female siblings were weaned onto either chow (2–3/sex/litter) or Caf (2–3/sex/litter) diet, matched on body weight. This formed four offspring groups: ChowChow, ChowCaf, CafChow, and CafCaf, with the first and second words denoting the maternal and post-weaning offspring diets, respectively. Rats were housed by sex, 2–4 per cage with littermates, where possible. Postweaning diets were continued for 11 weeks. 

### 4.5. Offspring Metabolic Measures

After weaning, offspring body weight was measured once weekly. At seven weeks of age, 24 h food intake was recorded. At eight weeks of age, rats underwent EchoMRI; at nine weeks, fasting glucose was measured from tail blood. Blood was centrifuged (5 min, 6000 RCF, 4 °C), and plasma was collected and stored at −30 °C for leptin, insulin, and triglyceride assays. 

### 4.6. Offspring Anxiety-Like Behaviour and Cognitive Measures

#### 4.6.1. Elevated Plus Maze (EPM)

At seven weeks of age, offspring were tested in the EPM, a plus shaped apparatus with two open (50 × 10 cm) and two closed (50 × 10 cm) arms, 50 cm off the floor. The test was conducted in a well-lit, quiet room with two floodlights on opposite sides, angled away from the maze. Each rat was placed in the centre of the maze facing an open arm and allowed to explore for 5 min. The number of arm entries and duration in each area was recorded by a camera located directly overhead and analysed by ANY-maze software.

#### 4.6.2. Place and Object Tests

Offspring underwent place and object recognition memory tests at 9–10 weeks of age. The apparatus was a black acrylic arena (60 × 60 × 60 cm). The objects used included drink bottles, coffee mugs, and cans, varying in size, material, and shape. All rats received a 10 min habituation session to the empty arena 24 h before their first test. On the first test day, half of the offspring in each group underwent the object task and half underwent the place task, and vice versa for the second day of testing. 

For both tasks, during the 5 min familiarisation phase, two identical objects were positioned in the middle squares of the arena. Then, the rat was returned to its home cage for 5 min and the arena and objects were cleaned with 50% ethanol. For the object test, the rat was placed back into the arena for 3 min with one familiar and one novel object in the same positions as the familiarisation phase. In the 3 min place test, the objects were the same as those used in familiarisation, but one was moved to a different location while the other remained in its original location. The ratio of time spent exploring the novel object/location and time spent exploring both objects was used as a measure of recognition. Data were scored using Macropod ODlog software by the experimenter and a second observer naïve to group allocation. 

### 4.7. Adult Plasma and Tissue Collection

At 14 weeks of age, 48 male offspring (ChowChow: *n* = 11, ChowCaf: *n* = 11, CafChow: *n* = 13, CafCaf: *n* = 13) and 46 female offspring (ChowChow: *n* = 11, ChowCaf: *n* = 11, CafChow: *n* = 11, CafCaf: *n* = 13) were anaesthetised by i.p. injection of ketamine/xylazine. Body weight, length, and girth (taken at the xiphoid process) were measured. After blood collection by cardiac puncture, rats were immediately decapitated. The liver was rapidly dissected and snap-frozen in liquid nitrogen and stored at −80 °C.

### 4.8. Plasma Hormone Measurements

Leptin and insulin plasma concentrations were measured by the enzyme linked immunosorbent assay (ELISA), according to the instructions in the kit (CrystalChem Inc., Chicago, IL, USA, Rat Leptin ELISA kit CAT#90040; Rat Insulin ELISA Kit CAT#90060), respectively. Leptin and insulin standard curves were prepared between 0.2–12.8 ng/mL and 0.1–12.8 ng/mL, respectively. Absorbance was read at 450 and 630 nm in a Versamax reader. Plasma triglycerides were measured in duplicate by a colorimetric method using triglyceride reagent (Roche, NSW, Australia) and standard (Sigma-Aldrich Pty Ltd., Macquarie Park, NSW, Australia).

A total of 200 µL reagent was added to each well (10 µL sample) and incubated at 37 °C for 5–10 min. Absorbance was read at 490 nm in a microplate reader.

### 4.9. Quantitative Real-Time Polymerase Chain Reaction (qRT-PCR)

#### 4.9.1. RNA Extraction and cDNA Synthesis

Aliquots of crushed liver tissue were combined with 1 mL Trizol (Sigma, St Louis, MO, USA) and then homogenised in a Precellys^®^ 24 (Bertin Technologies, Montigny-le-Bretonneux, France). Chloroform (200 µL) was added to each sample prior to centrifugation for 15 min (6000RCF, 4 °C). The upper aqueous layer (RNA) was transferred to a new microtube and precipitated with isopropanol, then washed with 75% ethanol and stored at −80 °C. RNA concentration was measured using a NanoDrop 1000 spectrophotometer (DeNovix). Approximately 2 μg RNA was reverse transcribed to cDNA using a high-capacity cDNA reverse transcription kit (Applied Biosystems, Foster city, CA, USA) and a PCR thermal cycler C1000 Bio-Rad-341. cDNA was diluted 1:10 and stored at −20 °C.

The stability of three housekeeper genes (hypoxanthine phosphoribosyl transferase 1 (HPRT1), TATA box binding protein (Tbp), and Beta-2-Microglobulin (B2M)) were evaluated in male and female offspring across treatment groups. RefFinder software [63] identified HPRT1 and B2M as the most stable genes.

#### 4.9.2. Real Time qPCR

A total of 1.5 μL of cDNA sample and 8.5 μL primer master mix were added to a 384 well plate in duplicate via the Eppendorf Epimotion^®^ 5075 robot (Eppendorf, AG, Hamburg, Germany). PCR was performed using the Quant Studio 12K Flex Real-time PCR system (Thermofisher scientific, Wilmington, DE, USA). Relative gene expression was conducted using the ∆∆CT method [64] with the geometric mean of the housekeepers. 

### 4.10. Data Analysis

Data were analysed using SPSS (IBM, v25). Data from dams were analysed by independent samples *t*-tests or by Mann–Whitney U-tests, where data failed assumptions of normality or homogeneity of variance tests. Offspring data were analysed in two- or three-way ANOVAs that included sex, maternal (Caf or chow), and post-weaning (Caf or chow) diet as between-subject factors, followed by post hoc pairwise comparisons applying the Tukey HSD correction. Two-way ANOVA (maternal diet × post weaning diet) was used for biochemical and gene expression analyses as males and females were processed on separate plates.

## 5. Conclusions

In summary, our results show that maternal obesity increased offspring adiposity in a sex-specific fashion. Weanling offspring born to Caf dams were smaller but had increased adiposity; by adulthood, only male offspring from Caf dams exhibited increased adiposity, liver mass, and body weight relative to chow dam counterparts. Male and female rat weanlings born to Caf dams had increased expression of hepatic ACC and FAS while in adulthood, we found decreased CPT-1 expression in male offspring. In conclusion, maternal and postweaning exposure to a palatable ‘cafeteria’ diet each impacted offspring metabolic health and their effects were largely independent, with greater impact in male than female offspring.

## Figures and Tables

**Table 1:** Litter characteristics.

		Maternal Diet	
		Chow (11)	Caf (9)
Litter size (n)	Total	14.72 ± 1.61	14.77 ± 2.90
	Female	7.18 ± 2.27	6.55 ± 2.24
	Male	7.54 ± 1.91	8.22 ± 1.71
Offspring body weight PND 1 (g)	Female	6.85 ± 0.41	6.61 ± 0.32
	Male	7.25 ± 0.44	6.81 ± 0.30 **

Litter size and offspring body weight at birth. Data were analysed by independent Student’s *t*-test. ** *p* < 0.01, significant effect of Caf diet.

**Table 2:** Maternal endpoint measures at three weeks post-partum.

	Chow (11)	Caf (9)
Body weight (g)	393.4 ± 7.2	393.8 ± 8.7
Nasoanal length (cm)	22.9 ± 0.2	23.1 ± 0.2
Girth (cm)	17.5 ± 0.2	17.7 ± 0.3
Liver weight (g)	18.04 ± 0.58	16.92 ± 0.48
RP fat weight (g)	2.93 ± 0.22	4.38 ± 0.56 *
Cecum (g)	7.77 ± 0.41	5.15 ± 0.78 *
Blood glucose (mmol/L)	9.69 ± 0.34	9.50 ± 0.53
Insulin (ng/mL)	0.75 ± 0.15	0.77 ± 0.10
Leptin (ng/mL)	1.98 ± 0.36	2.71 ± 0.53
Plasma triglyceride (nM)	2.30 ± 0.29	1.46 ± 0.30
Liver triglyceride (mg TG/mg tissue)	4.71 ± 0.48	7.02 ± 1.29

Body weight, anthropometric measures, liver, adipose, and cecum mass; blood glucose and plasma measures, and liver triglyceride content of chow and Caf females at three weeks post-partum. Data are expressed as mean ± SEM and were analysed by the independent Student’s *t*-test. * *p* < 0.05, significant effect of the Caf diet.

**Table 3:** Offspring endpoint measurements at PND 20.

	Male		Female	
Maternal Diet	Chow (*n* = 11)	Caf (*n* = 10)	Chow (*n* = 12)	Caf (*n* = 7)
Body weight (g) *	46.03 ± 2.72	42.14 ± 1.22	44.31 ± 1.78	39.83 ± 1.00
Nasoanal length (cm) *	10.95 ± 0.24	10.55 ± 0.18	10.79 ± 0.16	10.29 ± 0.08
Girth (cm)	8.45 ± 0.22	7.94 ± 0.25	8.54 ± 0.15	8.21 ± 0.12
Liver weight (g) *	1.74 ± 0.12	1.51 ± 0.07	1.65 ± 0.09	1.39 ± 0.06
RP fat weight (g) ** # ^	0.07 ± 0.01	0.17 ± 0.02	0.06 ± 0.01	0.09 ± 0.01
Kidney (g) *	0.26 ± 0.01	0.24 ± 0.01	0.27 ± 0.01	0.23 ± 0.01
Cecum (g)	0.35 ± 0.05	0.30 ± 0.02	0.28 ± 0.03	0.24 ± 0.02
Blood glucose (mmol/L) *	9.03 ± 0.37	8.38 ± 0.11	8.98 ± 0.24	8.29 ± 0.12

Body weight, nasoanal length, girth, liver weight, RP fat weight, cecum weight, and blood glucose concentrations of one representative male and female offspring per litter (where available) from chow and Caf females at PND 20. Data are expressed as mean ± SEM and were analysed by three-way ANOVA (maternal diet × sex). * *p* < 0.05, ** *p* < 0.01 significant effect of maternal diet; # *p* < 0.01 significant sex effect ^ *p* < 0.01 significant maternal diet × sex interaction.

**Table 4:** Offspring endpoint measures at 14 weeks.

	Male				Female			
Maternal Diet	Chow		Caf		Chow		Caf	
Offspring Diet	Chow 11	Caf 11	Chow 13	Caf 13	Chow 11	Caf 11	Chow 11	Caf 13
Body weight (g)	581.1 ± 12.8	724 ± 24.4 ††	619 ± 11.3 **	805.9 ± 14.4 †† **	309.4 ± 9.6	444.9 ± 27.8 ††	312.9 ± 10	433.7 ± 16.6 ††
Length (cm)	25.9 ± 0.2	26.9 ± 0.2 ††	26.2 ± 0.1	27.3 ± 0.2 ††	22.3 ± 0.2	23.2 ± 0.3 ††	22.2 ± 0.2	23 ± 0.16 ††
Tibia (cm)	4.31 ± 0.04	4.32 ± 0.04	4.33 ± 0.03	4.31 ± 0.04	3.77 ± 0.04	3.75 ± 0.04	3.72 ± 0.04	3.72 ± 0.04
Girth (cm)	20.4 ± 0.3	23.5 ± 0.5 ††	21.6 ± 0.3 *	24.5 ± 0.3 †† *	15.4 ± 0.3	19.3 ± 0.8 ††	16.1 ± 0.2	19.7 ± 0.5 ††
Liver (g)	20.74 ± 0.8	25.39 ± 1.4 ††	23.25 ± 0.5 *	28.68 ± 1.0 †† *	11.17 ± 0.4	13.56 ± 0.8 ††	10.36 ± 0.3	12.68 ± 0.4 ††
Left ventricle (g)	1.14 ± 0.06	1.33 ± 0.08 ††	1.11 ± 0.03	1.27 ± 0.03 ††	0.67 ± 0.03	0.88 ± 0.06 ††	0.67 ± 0.02	0.81 ± 0.03 ††
RP fat (g)	4.31 ± 0.4	12.96 ± 0.84 ††	5.52 ± 0.55 *	15.72 ± 0.80 †† *	2.64 ± 0.58	7.2 ± 0.77 ††	2.62 ± 0.37	6.14 ± 0.37 ††
Glucose (mmol/L)	11.7 ± 0.5	11.3 ± 0.7	11.5 ± 0.3	10.9 ± 0.3	9.6 ± 0.3	11.4 ± 0.3 ††	10.4 ± 0.4	11.8 ± 0.5 ††

Data are expressed as mean ± SEM of offspring at 14 weeks (n indicated above). Data were analysed by three-way ANOVA. Liver score was analysed using a non-parametric Kruskal–Wallis test. * *p* < 0.05, ** *p* < 0.01 main effect of maternal diet, †† *p* < 0.01 main effect of offspring postweaning diet.

## Data Availability

Data are available from the corresponding author on reasonable request.

## References

[1] Haththotuwa R.N., Wijeyaratne C.N., Senarath U. (2020). Worldwide epidemic of obesity. Obesity and Obstetrics.

[2] Hales C.M., Carroll M.D., Fryar C.D., Ogden C.L. *Prevalence of Obesity among Adults and Youth: United States, 2015–2016*. NCHS Data Brief. No. 288. 2017. https://stacks.cdc.gov/view/cdc/49223.

[3] Huse O., Hettiarachchi J., Gearon E., Nichols M., Allender S., Peeters A. (2018). Obesity in Australia. Obes. Res. Clin. Pract..

[4] Vahratian A. (2009). Prevalence of Overweight and Obesity Among Women of Childbearing Age: Results from the 2002 National Survey of Family Growth. Matern. Child Health J..

[5] Reiss K., Breckenkamp J., Borde T., Brenne S., David M., Razum O. (2015). Contribution of overweight and obesity to adverse preg-nancy outcomes among immigrant and non-immigrant women in Berlin, Germany. Eur. J. Public Health.

[6] Callaway L.K., Chang A.M., McIntyre H., Prins J. (2006). The prevalence and impact of overweight and obesity in an Australian obstetric population. Med. J. Aust..

[7] Lindam A., Johansson S., Stephansson O., Wikström A.-K., Cnattingius S. (2016). High Maternal Body Mass Index in Early Pregnancy and Risks of Stillbirth and Infant Mortality—A Population-Based Sibling Study in Sweden. Am. J. Epidemiol..

[8] Guelinckx I., Devlieger R., Beckers K., Vansant G. (2008). Maternal obesity: Pregnancy complications, gestational weight gain and nutrition. Obes. Rev..

[9] Catalano P.M., Ehrenberg H.M. (2006). Review article: The short- and long-term implications of maternal obesity on the mother and her offspring. BJOG Int. J. Obstet. Gynaecol..

[10] Green L.R., Hester R.L. (2016). Parental Obesity: Intergenerational Programming and Consequences.

[11] Voerman E., Santos S., Golab B.P., Amiano P., Ballester F., Barros H., Bergström A., Charles M.-A., Chatzi L., Chevrier C. (2019). Maternal body mass index, gestational weight gain, and the risk of overweight and obesity across childhood: An individual participant data meta-analysis. PLoS Med..

[12] Schack-Nielsen L., Michaelsen K.F., Gamborg M., Mortensen E.L., Sørensen T.I.A. (2010). Gestational weight gain in relation to offspring body mass index and obesity from infancy through adulthood. Int. J. Obes..

[13] Fraser A., Tilling K., Macdonald-Wallis C., Sattar N., Brion M.-J., Benfield L., Ness A., Deanfield J., Hingorani A., Nelson S.M. (2010). Association of Maternal Weight Gain in Pregnancy With Offspring Obesity and Metabolic and Vascular Traits in Childhood. Circulation.

[14] Dello Russo M., Ahrens W., De Vriendt T., Marild S., Molnar D., Moreno L.A., Reeske L.A., Veidebaum T., Kourides Y.A., Barba G. (2013). Gestational weight gain and adiposity, fat distribution, metabolic profile, and blood pressure in offspring: The IDEFICS project. Int. J. Obes..

[15] Godfrey K.M., Reynolds R.M., Prescott S.L., Nyirenda M., Jaddoe V.W.V., Eriksson J.G., Broekman B.F.P. (2017). Influence of maternal obesity on the long-term health of offspring. Lancet Diabetes Endocrinol..

[16] Reynolds C.M., Segovia S.A., Vickers M.H. (2017). Experimental Models of Maternal Obesity and Neuroendocrine Programming of Metabolic Disorders in Offspring. Front. Endocrinol..

[17] Zambrano E., Nathanielsz P.W. (2013). Mechanisms by which maternal obesity programs offspring for obesity: Evidence from animal studies. Nutr. Rev..

[18] Ribaroff G.A., Wastnedge E., Drake A.J., Sharpe R.M., Chambers T.J.G. (2017). Animal models of maternal high fat diet exposure and effects on metabolism in offspring: A meta-regression analysis. Obes. Rev..

[19] Chen H., Morris M.J. (2009). Differential Responses of Orexigenic Neuropeptides to Fasting in Offspring of Obese Mothers. Obesity.

[20] Bahari H., Caruso V., Morris M.J. (2013). Late-Onset Exercise in Female Rat Offspring Ameliorates the Detrimental Metabolic Impact of Maternal Obesity. Endocrinology.

[21] Kjaergaard M., Nilsson C., Rosendal A., Nielsen M.O., Raun K. (2014). Maternal chocolate and sucrose soft drink intake induces hepatic steatosis in rat offspring associated with altered lipid gene expression profile. Acta Physiol..

[22] Bayol S.A., Simbi B.H., Fowkes R.C., Stickland N.C. (2010). A maternal “junk food” diet in pregnancy and lactation promotes nonal-coholic fatty liver disease in rat offspring. Endocrinology.

[23] Dahlhoff M., Pfister S., Blutke A., Rozman J., Klingenspor M., Deutsch M., Rathkolb B., Fink B., Gimpfl M., de Angelis M.H. (2014). Peri-conceptional obesogenic exposure induces sex-specific programming of disease susceptibilities in adult mouse offspring. Biochim. Biophys. Acta (BBA)-Mol. Basis Dis..

[24] Hasebe K., Kendig M.D., Morris M.J. (2021). Mechanisms Underlying the Cognitive and Behavioural Effects of Maternal Obesity. Nutrients.

[25] Sanchez C.E., Barry C., Sabhlok A., Russell K., Majors A., Kollins S.H., Fuemmeler B.F. (2018). Maternal pre-pregnancy obesity and child neuro-developmental outcomes: A meta-analysis. Obes Rev..

[26] Jo H., Schieve L.A., Sharma A.J., Hinkle S.N., Li R., Lind J.N. (2015). Maternal Prepregnancy Body Mass Index and Child Psychosocial Development at 6 Years of Age. Pediatrics.

[27] Contu L., Hawkes C.A. (2017). A review of the impact of maternal obesity on the cognitive function and mental health of the off-spring. Int. J. Mol. Sci..

[28] Sasaki A., de Vega W., Sivanathan S., St-Cyr S., McGowan P. (2014). Maternal high-fat diet alters anxiety behavior and glucocorticoid signaling in adolescent offspring. Neuroscience.

[29] Sasaki A., de Vega W.C., St-Cyr S., Pan P., McGowan P. (2013). Perinatal high fat diet alters glucocorticoid signaling and anxiety behavior in adulthood. Neuroscience.

[30] Graf A.E., Lallier S.W., Waidyaratne G., Thompson M.D., Tipple T.E., Hester M.E., Trask A.J., Rogers L.K. (2016). Maternal high fat diet exposure is associated with increased hepcidin levels, decreased myelination, and neurobehavioral changes in male offspring. Brain Behav. Immun..

[31] Zieba J., Uddin G.M., Youngson N.A., Karl T., Morris M.J. (2019). Long-term behavioural effects of maternal obesity in C57BL/6J mice. Physiol. Behav..

[32] O’Reilly J.R.O., Reynolds R.M. (2012). The risk of maternal obesity to the long-term health of the offspring. Clin. Endocrinol..

[33] Andres A., Hull H.R., Shankar K., Casey P.H., Cleves M.A., Badger T.M. (2015). Longitudinal body composition of children born to mothers with normal weight, overweight, and obesity. Obesity.

[34] Rajasingam D., Seed P.T., Briley A.L., Shennan A.H., Poston L. (2009). A prospective study of pregnancy outcome and biomarkers of oxidative stress in nulliparous obese women. Am. J. Obstet. Gynecol..

[35] Pomar C.A., Van Nes R., Sánchez J., Picó C., Keijer J., Palou A. (2017). Maternal consumption of a cafeteria diet during lactation in rats leads the offspring to a thin-outside-fat-inside phenotype. Int. J. Obes..

[36] Mucellini A.B., Goularte J.F., Da Cunha A.C.D.A., Caceres R.C., Noschang C., Da Silva Benetti C., Silveira P.P., Sanvitto G.L. (2014). Effects of exposure to a cafeteria diet during gestation and after weaning on the metabolism and body weight of adult male offspring in rats. Br. J. Nutr..

[37] Rolls B.J., Rowe E.A. (1982). Pregnancy and lactation in the obese rat: Effects on maternal and pup weights. Physiol. Behav..

[38] Sánchez J., Priego T., Palou M., Tobaruela A., Palou A., Pico C. (2008). Oral Supplementation with Physiological Doses of Leptin During Lactation in Rats Improves Insulin Sensitivity and Affects Food Preferences Later in Life. Endocrinology.

[39] Ong Z.Y., Muhlhausler B.S. (2011). Maternal “junk-food” feeding of rat dams alters food choices and development of the mesolimbic reward pathway in the offspring. FASEB J..

[40] McDonald S.D., Han Z., Mulla S., Beyene J., Knowledge Synthesis Group (2010). Overweight and obesity in mothers and risk of preterm birth and low birth weight infants: Systematic review and meta-analyses. BMJ.

[41] Drake A.J., Reynolds R.M. (2010). Focus on obesity: Impact of maternal obesity on offspring obesity and cardiometabolic disease risk. Reproduction.

[42] Francis E.C., Dabelea D., Shankar K., Perng W. (2021). Maternal diet quality during pregnancy is associated with biomarkers of met-abolic risk among male offspring. Diabetologia.

[43] Rajia S., Chen H., Morris M.J. (2010). Maternal overnutrition impacts offspring adiposity and brain appetite markers-modulation by postweaning diet. J. Neuroendocr..

[44] Litzenburger T., Huber E.-K., Dinger K., Wilke R., Vohlen C., Selle J., Kadah M., Persigehl T., Heneweer C., Dötsch J. (2020). Maternal high-fat diet induces long-term obesity with sex-dependent metabolic programming of adipocyte differentiation, hypertrophy and dysfunction in the offspring. Clin. Sci..

[45] Riant E., Waget A., Cogo H., Arnal J.-F., Burcelin R., Gourdy P. (2009). Estrogens Protect against High-Fat Diet-Induced Insulin Resistance and Glucose Intolerance in Mice. Endocrinology.

[46] Bryzgalova G., Gao H., Ahren B., Zierath J.R., Galuska D., Steiler T.L., Dahlman-Wright K., Nilssosn S., Gustafsson J.A., Efendic S. (2006). Evidence that oestrogen receptor-α plays an important role in the regulation of glucose homeostasis in mice: Insulin sensitivity in the liver. Diabetologia.

[47] Shrestha N., Ezechukwu H.C., Holland O.J., Hryciw D.H. (2020). Developmental programming of peripheral diseases in offspring exposed to maternal obesity during pregnancy. Am. J. Physiol. Integr. Comp. Physiol..

[48] Dudele A., Hougaard K.S., Kjolby M., Hokland M., Winther G., Elfving B., Wegener G., Nielsen A.L., Larsen A., Nøhr M.K. (2017). Chronic maternal inflammation or high-fat-feeding programs offspring obesity in a sex-dependent manner. Int. J. Obes..

[49] Kruse M., Seki Y., Vuguin P.M., Du X.Q., Fiallo A., Glenn A.S., Singer S., Breuhahn K., Katz E.B., Charron M.J. (2013). High-Fat Intake During Pregnancy and Lactation Exacerbates High-Fat Diet-Induced Complications in Male Offspring in Mice. Endocrinology.

[50] Parente L.B., Aguila M.B., Mandarim-De-Lacerda C.A. (2008). Deleterious effects of high-fat diet on perinatal and postweaning periods in adult rat offspring. Clin. Nutr..

[51] Ruager-Martin R., Hyde M.J., Modi N. (2010). Maternal obesity and infant outcomes. Early Hum. Dev..

[52] Bringhenti I., Ornellas F., Martins M.A., Mandarim-De-Lacerda C., Aguila M.B. (2015). Early hepatic insult in the offspring of obese maternal mice. Nutr. Res..

[53] Ornellas F., Souza-Mello V., Mandarim-De-Lacerda C.A., Aguila M.B. (2015). Programming of Obesity and Comorbidities in the Progeny: Lessons from a Model of Diet-Induced Obese Parents. PLoS ONE.

[54] Giudetti A.M., Micioni Di Bonaventura M.V., Ferramosca A., Longo S., Micioni Di Bonaventura E., Friuli M., Romano A., Gaetani S., Cifani C. (2020). Brief daily access to cafeteria-style diet impairs hepatic metabolism even in the absence of excessive body weight gain in rats. FASEB J..

[55] Daniel Z.C., Akyol A., McMullen S., Langley-Evans S.C. (2014). Exposure of neonatal rats to maternal cafeteria feeding during suckling alters hepatic gene expression and DNA methylation in the insulin signalling pathway. Genes Nutr..

[56] Bruce K.D., Cagampang F.R., Argenton M., Zhang J., Ethirajan P.L., Burdge G.C., Bateman A.C., Clough G.F., Poston L., Hanson M.A. (2009). Maternal high-fat feeding primes steatohepatitis in adult mice offspring, involving mitochondrial dysfunction and altered lipogenesis gene expression. Hepatology.

[57] Wright T., Langley-Evans S., Voigt J.-P. (2011). The impact of maternal cafeteria diet on anxiety-related behaviour and exploration in the offspring. Physiol. Behav..

[58] Speight A., Davey W.G., McKenna E., Voigt J.-P.W. (2016). Exposure to a maternal cafeteria diet changes open-field behaviour in the developing offspring. Int. J. Dev. Neurosci..

[59] Curi H.T., Dias C.T., da Luz Camargo M.L.M., dos Santos Gomez P., Gomes M.F.P., Beserra-Filho J.I.A., Medeiros A., Ribeiro A.M., Simabuco F.M., Lambertucci R.H. (2021). Maternal high-fat diet increases anhedonic behavior and modulates hippocampal Mash1 and BDNF expression in adult offspring. Neurosci. Lett..

[60] Clark T.D., Crean A.J., Senior A.M. (2021). Obesogenic diets induce anxiety in rodents: A systematic review and meta-analysis. Obes. Rev..

[61] Cordner Z.A., Khambadkone S.G., Boersma G.J., Song L., Summers T.N., Moran T.H., Tamashiro K.L. (2019). Maternal high-fat diet results in cog-nitive impairment and hippocampal gene expression changes in rat offspring. Exp. Neurol..

[62] Leigh S.-J., Kendig M.D., Morris M.J. (2019). Palatable Western-style Cafeteria Diet as a Reliable Method for Modeling Diet-induced Obesity in Rodenfts. J. Vis. Exp..

[63] Xie F., Xiao P., Chen D., Xu L., Zhang B. (2012). miRDeepFinder: A miRNA analysis tool for deep sequencing of plant small RNAs. Plant Mol. Biol..

[64] Livak K.J., Schmittgen T.D. (2001). Analysis of relative gene expression data using real-time quantitative PCR and the 2- ΔΔCT method. Methods.

